# The impact of coping styles on the mental health of healthcare workers one year after the initial COVID-19 outbreak in China

**DOI:** 10.1192/j.eurpsy.2023.1242

**Published:** 2023-07-19

**Authors:** N. Haghbin, M. J. Gonzalez Mendez

**Affiliations:** 1 Medicine and Health, University of Sydney, Sydney, Australia; 2 Health Ciences Institute, Universidad de O’Higgins, Rancagua, Chile

## Abstract

**Introduction:**

A 2020 study in Wuhan residents reported 70.2% of participants faced the COVID-19 pandemic using active coping strategies, and we wanted to explore its comparability in Chinese Health Care Workers (HCWs) across 7 regions in China 1 year after the initial outbreak.

**Objectives:**

The study analyzed coping strategies utilized by different Chinese HCWs under a stressful period like the COVID-19 pandemic and three psychological scales were used to assess its effect on five psychological outcomes such as depression, anxiety, stress, post-traumatic stress disorder and suicidal ideation.

**Methods:**

A cross-sectional self-administered online questionnaire was conducted during the period of November 2020 and March 2021 and included sociodemographic information, work environment before and during the pandemic, experiences, fears and concerns about COVID-19 and three psychological scales including Depression, Anxiety and Stress Scale-21(DASS-21), Primary Care PTSD Screen for DSM-5 (PC-PTSD-5) and Simplified Coping Style Questionnaire (SCSQ-20). Chi-square analysis was used to explore categorical association.

**Results:**

The findings demonstrated that 633 (52.5%) of the participants used passive coping strategies, while 600 (47.5%) used active coping strategies. Passive coping strategies with at least one mental health problem were positively correlated with participants having a previous chronic disease diagnosis, working days in a week during the outbreak, PPE availability, days in isolation for being suspected or a confirmed case of COVID-19, worries about infecting relatives and the pandemic affecting family’s financial situation.

**Conclusions:**

Developing and creating intervention programs to strengthen active coping strategies will improve mental health outcomes in Chinese HCWs during the COVID pandemic.

**Disclosure of Interest:**

None Declared

